# Successful treatment of concomitant alopecia universalis and Crohn’s disease with upadacitinib: A case report

**DOI:** 10.1177/2050313X231160914

**Published:** 2023-03-21

**Authors:** Leah A Johnston, Cathy Lu, Susan M Poelman

**Affiliations:** 1 Health Sciences Centre, Cumming School of Medicine, University of Calgary, Calgary, AB, Canada; 2 Division of Gastroenterology, Department of Medicine, University of Calgary, Calgary, AB, Canada; 3 Beacon Dermatology, Calgary, AB, Canada

**Keywords:** Alopecia areata, upadacitinib, JAK inhibitor, hair loss, inflammatory bowel disease, Crohn’s disease

## Abstract

Alopecia areata is a non-scarring, autoimmune hair loss disorder that is associated with inflammatory bowel disease. Alopecia areata and inflammatory bowel disease may have a common pathogenic mechanism that involves the Janus kinase/STAT pathway. In addition, there are previous case reports of patients who developed alopecia areata while on biologic therapies for inflammatory bowel disease. JAK1 inhibitors are currently undergoing investigation as potential therapies for Crohn’s disease. Upadacitinib, an oral JAK1 inhibitor, has demonstrated efficacy in treating Crohn’s disease during phase III clinical trials. In this case report, we present a 23-year-old man with Crohn’s disease who previously developed alopecia areata while on adalimumab. He had near-complete resolution of his alopecia universalis after 7 months of treatment with upadacitinib while on concurrent ustekinumab for Crohn’s disease, which he had been taking for 16 months prior to starting upadacitinib. Upadacitinib may be a beneficial therapy for treating concomitant alopecia areata and Crohn’s disease.

## Introduction

Alopecia areata (AA) is a non-scarring, autoimmune hair loss disorder that involves CD8^+^ T lymphocyte-mediated disruption of the hair follicle bulb and is characterized by circular patches of hair loss.^1^ In severe cases, AA can progress to alopecia universalis, which presents with total body hair loss. The pathogenesis of AA involves production of interferon (IFN)-γ by CD8^+^ T lymphocytes, leading to release of interleukin (IL)-2, IL-7, IL-15, and IL-21 and subsequent activation of the JAK (Janus kinase)/STAT signaling pathway.^1,2^ Recently, clinical trials of JAK inhibitors have demonstrated the efficacy of tofacitinib and ruxolitinib in treating AA and in 2022, oral baricitinib received Food and Drug Administration (FDA) approval for use in severe AA.^3,4^

JAK inhibitors have also shown promise in treating inflammatory bowel disease (IBD).^5^ In 2018, tofacitinib, a non-selective JAK inhibitor, was approved by the FDA for treatment of ulcerative colitis.^5^ However, clinical trials of tofacitinib produced conflicting results in Crohn’s disease (CD). Selective oral JAK1 inhibitors, such as filgotinib and upadacitinib, have demonstrated greater efficacy in CD management during phase II and III randomized controlled trials, respectively.^6,7^ Upadacitinib maintenance trials have shown that a significantly greater number of CD patients achieved clinical remission, as well as endoscopic response and remission after 52 weeks on upadacitinib 15 or 30 mg, compared to placebo.^7^

A systematic review found that the prevalence of AA in individuals with IBD was 0.63%, which was significantly higher than the general population risk.^8^ Both CD and AA have autoimmune, T-cell mediated mechanisms that involve activation of the JAK/STAT pathway.^2,5^ While upadacitinib has not been investigated in clinical trials for AA, there have been case reports of concomitant AA improving in patients who were treated with upadacitinib for atopic dermatitis.^9–11^ In this case report, we describe a patient with concomitant AA and CD who was successfully treated with upadacitinib.

## Case report

A 23-year-old Caucasian man was diagnosed with CD at the age of 17 years, following a 2-month history of abdominal pain, diarrhea, and weight loss. He was initially treated with a tapering course of corticosteroids and then started treatment with adalimumab at 40 mg q2 weeks, with subsequent dose escalation 9 months later for subtherapeutic levels of 7.7 µg/mL. After 6 months on adalimumab, he developed a circular patch of hair loss on the occipital scalp hairline. He also developed hair loss on the arms and legs. He was diagnosed with AA and the patch of hair loss on his scalp was subsequently treated with intralesional triamcinolone acetonide injections, to which he had a suboptimal response.

**Figure 1.**
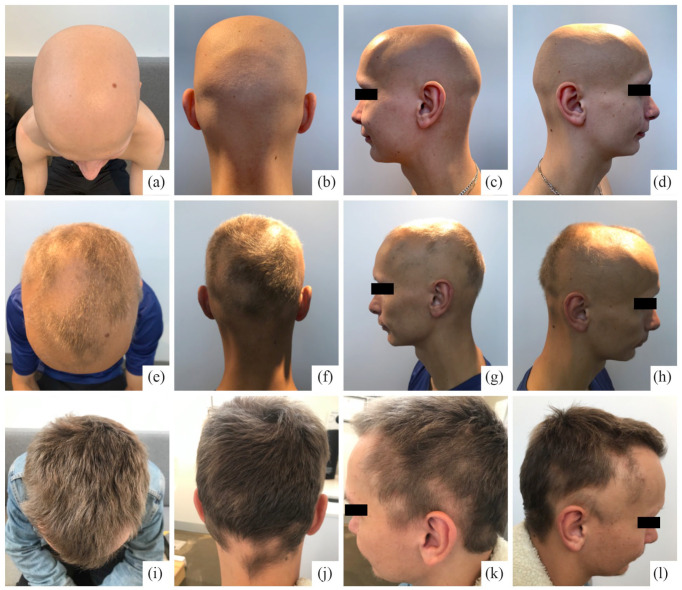
Clinical course of alopecia areata on the scalp at baseline (a, b, c, d) and following daily treatment with upadacitinib 30 mg at 4 months (e, f, g, h) and 7 months (i, j, k, l). At 4 months, partial hair regrowth on the scalp was evident. Near-complete regrowth of scalp hair from alopecia universalis at baseline was observed after 7 months, with only a few remaining circular patches of hair loss with partial regrowth on the temples and occipital scalp hairline.

At 3.5 years since his initial CD diagnosis, he was admitted to hospital for a CD flare-up. His adalimumab was switched to ustekinumab 90 mg q8 weeks. He presented to our dermatology clinic 1 month later with increasing hair loss on the scalp, arms, and legs. He was prescribed a 3-week tapering course of prednisone 40 mg and oral methotrexate 20 mg weekly for 6 weeks. After a few months, he noticed some hair regrowth but continued to develop new patches of hair loss on the scalp, arms, legs, chest, back, and eyebrows. His ustekinumab dose was escalated to q4 weeks and 5 months later, and he had developed alopecia universalis. After another 5 months, he started upadacitinib 30 mg daily for AA, in addition to continuing ustekinumab q4 weeks. At 4 months, he had significant partial hair regrowth on the scalp. By 7 months since initiation of upadacitinib, he had near-complete resolution of AA on the scalp (Figure 1), with only a few remaining patches of hair loss on the temples and occipital scalp hairline. He also had significant hair regrowth on the eyebrows, arms, and legs. In addition, his abdominal magnetic resonance imaging (MRI) showed minimal CD activity, and his CD was confirmed to be in clinical remission by his gastroenterologist.

## Discussion

In this case, upadacitinib was successful in treating AA in a patient with CD who developed non-response to adalimumab. Although biologic medications that target individual inflammatory cytokines are often highly effective in managing IBD, loss of efficacy is common.^12^ JAK inhibitors may be beneficial in these patients due to their direct effects on inhibiting the JAK/STAT pathway, which is induced by multiple different inflammatory cytokines.^12^

Potential triggers for AA include psychological or physiological stress, recent viral illnesses, and medications.^1^ AA has been previously reported in individuals who were treated with tumor necrosis factor (TNF)-α inhibitors, including adalimumab.^13–16^ Both adalimumab and CD itself may have precipitated AA in our patient. Notably, he developed rapid progression of AA after switching from adalimumab to ustekinumab for his CD. Three previous cases have been reported of AA developing in patients on ustekinumab therapy for psoriasis.^17,18^ However, other case reports have demonstrated improvement of AA with ustekinumab.^19–21^ One retrospective study of ustekinumab in patients with previous adverse reactions to TNF-α inhibitors found that two patients who developed AA had complete resolution of hair loss after switching to ustekinumab.^21^

There are two previous case reports that showed improvement of both AA and CD with oral JAK inhibitors.^22,23^ The first patient was a 37-year-old woman who developed AA after 2 years of treatment for CD with adalimumab.^22^ After 5 months on tofacitinib 15 mg and 8 weeks of oral budesonide 9 mg, she demonstrated complete hair regrowth on the scalp and improvement of CD symptoms. The second patient was a 36-year-old woman who developed CD prior to the onset of AA.^23^ She was treated with an investigational JAK inhibitor for 6 months, followed by tofacitinib 5 mg BID for 10 months, after which she had complete resolution of AA and control of CD.

Four previous case reports, which included five patients, have been published on treatment of AA with upadacitinib.^9–11,24^ All five patients had substantial improvement or total resolution of AA with upadacitinib 30 mg daily. In four patients, the primary indication for treatment was atopic dermatitis and resolution of AA was an unexpected but beneficial secondary outcome. Upadacitinib may be a worthwhile therapy to study in future clinical trials for AA.

In conclusion, this is the first case report showing improvement of both alopecia universalis and CD with upadacitinib. Drug-induced AA is a rare but psychologically devastating complication for patients on biologic therapies for CD, which was observed in our patient during treatment with adalimumab and ustekinumab. This case demonstrates that upadacitinib is an important consideration for treatment of AA in patients with IBD.
